# Double-Stranded RNA Uptake through Topical Application, Mediates Silencing of Five CYP4 Genes and Suppresses Insecticide Resistance in *Diaphorina citri*

**DOI:** 10.1371/journal.pone.0110536

**Published:** 2014-10-20

**Authors:** Nabil Killiny, Subhas Hajeri, Siddharth Tiwari, Siddarame Gowda, Lukasz L. Stelinski

**Affiliations:** 1 Department of Entomology and Nematology, Citrus Research and Education Center, IFAS, University of Florida, Lake Alfred, Florida, United States of America; 2 Department of Plant Pathology, Citrus Research and Education Center, IFAS, University of Florida, Lake Alfred, Florida, United States of America; New Mexico State University, United States of America

## Abstract

Silencing of genes through RNA interference (RNAi) in insects has gained momentum during the past few years. RNAi has been used to cause insect mortality, inhibit insect growth, increase insecticide susceptibility, and prevent the development of insecticide resistance. We investigated the efficacy of topically applied dsRNA to induce RNAi for five Cytochrome P_450_ genes family 4 (*CYP4*) in *Diaphorina citri*. We previously reported that these *CYP4* genes are associated with the development of insecticide resistance in *D. citri*. We targeted five *CYP4* genes that share a consensus sequence with one dsRNA construct. Quantitative PCR confirmed suppressed expression of the five *CYP4* genes as a result of dsRNA topically applied to the thoracic region of *D. citri* when compared to the expression levels in a control group. Western blot analysis indicated a reduced signal of cytochrome P_450_ proteins (45 kDa) in adult *D. citri* treated with the dsRNA. In addition, oxidase activity and insecticide resistance were reduced for *D. citri* treated with dsRNA that targeted specific *CYP4* genes. Mortality was significantly higher in adults treated with dsRNA than in adults treated with water. Our results indicate that topically applied dsRNA can penetrate the cuticle of *D. citri* and induce RNAi. These results broaden the scope of RNAi as a mechanism to manage pests by targeting a broad range of genes. The results also support the application of RNAi as a viable tool to overcome insecticide resistance development in *D. citri* populations. However, further research is needed to develop grower-friendly delivery systems for the application of dsRNA under field conditions. Considering the high specificity of dsRNA, this tool can also be used for management of *D. citri* by targeting physiologically critical genes involved in growth and development.

## Introduction

RNA interference (RNAi) is a promising tool for studying functional genomics in eukaryotes and insects in particular [1], [2]. Anti-sense (nonsense) RNA strand transcription has been used for over three decades to inhibit gene activity [3]. The efficacy of anti-sense silencing depends on hybridization between the injected RNA and an endogenous messenger. Since the discovery of double-stranded RNA (dsRNA) mediated gene-specific silencing in the nematode, *Caenorhabditis elegans* (Maupas) [4], dsRNA-mediated RNAi has been employed with various insects to silence specific genes [2]. RNAi has been widely used in various insect orders, including Coleoptera, Dictyoptera, Diptera, Hemiptera, Hymenoptera, Isoptera, Lepidoptera, Neuroptera, and Orthoptera [5], [6], [7]. The systemic nature of dsRNA-mediated RNAi has allowed this tool to be used in the management of various insect pests [8]–[13].

The cytochrome P_450_ monooxygenases are an important group of enzymes that are involved in the metabolism of xenobiotic compounds in insects. This group of enzymes is associated with insecticide resistance and metabolism of a wide range of endogenous and exogenous compounds that includes hormones, pheromones, insecticides, and plant secondary compounds in insects [14]–[18]. Overtranscription of families 4, 6, 9, and 12 has been frequently linked to insecticide metabolism and resistance [19]–[22].

The Asian citrus psyllid, *Diaphorina citri* Kuwayama (Hemiptera: Psyllidae), is perhaps the most destructive pest of citrus, mainly because it is a vector for the putative causal agent of huanglongbing (HLB), *Candidatus* Liberibacter asiaticus (*C*Las) [23]. HLB is a deadly citrus disease with no known cure [23], [24]. Currently, the main tools that limit the spread of the disease are insecticides to manage the vector [18], [25], [26]. *D. citri* are susceptible to several insecticide classes, which includes the pyrethroids, organophosphates, carbamates, neonicotinoids, insect growth regulators, horticultural oils, and lipid synthesis inhibitors [27]. Foliar treatments may suppress populations for 3 weeks following application [27]. Broad-spectrum insecticides (pyrethroids, organophosphates, and neonicotinoids) are more effective against *D. citri* than IGRs or oils, and insecticide use against *D. citri* is most effective when populations are not actively reproducing [28]. Systemic soil-applied insecticides provide a much longer duration of population control (months) than foliar insecticides (weeks) [27]. The neonicitonoids have been the main class of effective systemic insecticides for *D. citri* control during the past decade [27]. Systemic neonicotinoids are particularly effective in protecting young trees as they mature into production [29].

Intense insecticide use has led to the development of varying levels of insecticide resistance in populations of *D. citri* in Florida, USA [26]. This is particularly concerning for the neonicotinoid class, since these are the main current tools for protecting young trees from *C*Las infection [29]. A metabolic mechanism for the evolution of insecticide resistance in populations of *D. citri*, particularly for neonicotinoids, is supported by increased activities of detoxifying enzymes and overexpression of Cytochrome P_450_ genes family 4 (*CYP4*) [17], [21], [26], [30], [31]. In the present study, we targeted the abovementioned *CYP4* genes for silencing by topical application of specific dsRNA to the thorax of newly emerged *D. citri* adults. Additionally, we tested the effect of dsRNA treatment on insecticide resistance by comparing mortality of known susceptible and resistant populations of *D. citri*.

## Materials and Methods

### Insect populations

A laboratory susceptible population (LS) of *D. citri* was maintained in a greenhouse at the Citrus Research and Education Center, Lake Alfred, Florida. The culture was established in 2000 using field populations from Polk County, Florida and maintained on sweet orange (*Citrus sinensis* (L.) Osbeck) without exposure to insecticides in a greenhouse at 27–28°C, with 60–65% relative humidity and a 14∶10 (light:dark) photocycle hours. Three field populations of *D. citri* were collected from commercial citrus groves in Florida during 2013. The populations were collected with permissions from private groves. Name of groves, counties, and GPS coordinates are as the following: *i*) GapWay Groves (Private managed grove), Polk County (PL) (28° 05′ 40.14″ N; 81° 43′ 19.03″ W); *ii*) Winter Garden, Conserve II (Private managed grove), Lake County (LA) (28° 27′ 52.17″ N; 81° 39′ 31.69″ W); and *iii)* Uncle Matt’s Organic (Organically managed grove), Lake County (OG) (28° 31′ 00.88″ N; 81° 40′ 01.90″ W). Adults were collected using sweep nets and aspirators, transferred to the laboratory, released onto citrus plants within Plexiglas cages (40×40×40 cm), and used in bioassays shortly thereafter.

### Constructing dsRNA

A consensus sequence, derived from five previously published *CYP4* sequences [21], was used to design *CYP4*-specific primers (Table 1). The *CYP4*-specific primers were tailed with a T7 promoter sequence to generate sense and antisense transcripts separately.

**Table 1:** Primers used in this investigation.

Purpose	Gene	Sequence	Reference
*ds-RNA synthesis*			
	**CYP4**	Forward CACG*ttaattaa* ACGTTCATGTTCGAGGGGCACGATACAACAAC	This
	**Sense**	Reverse GAGC*aggcct*GAAGGGTACATAGGAGTAAGGATGACGTTTCTG	investigation
	**CYP4 Antisense**	Forward GCAGCA*TAATACGACTCACTATAGGGAGA*GAAGGGTACATAGGAGTAAGGATGACGTTTCTG Reverse ACGTTCATGTTCGAGGGGCACGATACAACAAC	This investigation
	**GFP**	Forward GCGAACTAGTATGGCTAGCAAAGGAGAAGAACTTTTCACTG	El-Shesheny
		Reverse GAGACCGCGGCTACCCCTCGAGTTATTTGTAGAGCTCATC	et al. [32]
*Gene expression*			
	*CYP4 genes*		
	CYP4C67	Forward TGGAACGTGTCATCAAGGAG	Tiwari et al.
		Reverse CCGGATTGAAACTGTTAGGC	[21]
	CYP4DA1	Forward AGTGGTGTCGGAAATTGAGG	
		Reverse GTTCGAGCCACCTGGAGATA	
	CYPC68	Forward CTAGCCTGGACCCTCTTCCT	
		Reverse ACCCTCCCTATGAACGGAAC	
	CYPG70	Forward GCCGGAAGTTCTTTCTTCCT	
		Reverse TAACGGGTACTGGTGGGAAC	
	CYPDB1	Forward CTGTACGCTCTGGGACATCA	
		Reverse TTGAGCGGTGCATAGAGTTG	
	*Reference genes*		
	α-tubulin	Forward CAGGTCTTGTGTGGGACGTA	Tiwari et al. [21]
		Reverse GGCCACAGTTTGTTTCTTGC	
	Actin	Forward CCCTGGACTTTGAACAGGAA	
		Reverse CTCGTGGATACCGCAAGATT	

Total RNA isolation was performed on groups of 40–50 psyllids using the SV total RNA isolation kit (Promega, Madison, WI, USA). One microgram of RNA was used to synthesize cDNA using the *CYP4*-specific reverse primers and iScript cDNA synthesis kit (Bio-Rad, Hercules, CA, USA). Sense and antisense PCR products were generated in separate PCR reaction using specific combination of primers (Table 1). To generate plus-sense transcripts, sense primer with T7 promoter sequence and regular antisense primer were used; while to generate antisense transcripts, regular sense primer and antisense primer with T7 promoter sequence were used. Sense and antisense transcripts were annealed by denaturing at 70°C for 10 min, followed by slowly cooling to room temperature for 20 min. To eliminate DNA template and single-stranded RNA, dsRNA was treated with DNase I and RNase A. The dsRNA was then purified of proteins and free nucleotides using the phenol-chloroform purification method. The amount of purified dsRNA was measured with a NanoDrop Spectrophotometer. We used dsRNA-*gfp* as an irrelevant dsRNA (control). dsRNA-*gfp* was produced as described above. Green fluorescent protein (GFP) mRNA is 732 bp in length. Specific primers (Table 1) were used to amplify the full-length of GFP gene by using TMV-30BGFP according to El-Shesheny et al. [32].

### *D. citri* treatment with dsRNA

Purified dsRNA was serially diluted using RNase-free water to obtain desired concentrations of dsRNA. Three concentrations of dsRNA (50, 75, and 100 ng/µl) and a control (0 ng/µl) were used to treat *D. citri* adults. *D. citri* adults were anaesthetized under CO_2_ within a few hours of eclosion. A 0.2 µl droplet containing10, 15, or 20 ng of dsRNA was topically applied to the ventral side of the thorax using a 10 µl Hamilton syringe. To investigate the effect of dsRNA- *P_450_* on gene and protein expression and enzymatic activity, treated adults were placed into 60 mm plastic disposable Petri dishes that were lined with citrus leaf disks, as a food source, over agar beds as described in Tiwari et al. [17]. Petri dishes with treated adults were kept at 25±1°C and 50±5% RH, with a 14∶10 h light:dark photoperiod, in a growth chamber for 72 h. Insects were collected and stored in −20°C until use. dsRNA-*gfp* was used as a non-relevant dsRNA control.

### Cytochrome *P_450_* (general oxidase) assay

The activity of cytochrome *P_450_* was quantified and expressed in terms of general oxidase level, which is an indirect measure of cytochrome *P_450_* by using heme peroxidation as described in Tiwari et al. [31]. This method has been considered a reliable tool for comparing differences in general oxidase levels based on hemoprotein levels. Because heme constitutes the majority of cytochrome *P_450_* in non-blood-fed insects, quantification of heme activity has been used to compare the levels of cytochrome *P_450_* on the basis of general oxidase levels [33]. In brief, heme peroxidase activity was measured using 3,3′5,5′-tetra-methylbenzidine (TMBZ) (Sigma Aldrich) as the substrate. Five replicates, each consisting of three insects, were performed for each treatment.

### Gene expression analysis

Live adult *D. citri* from each treatment were subjected to RNA isolation and cDNA synthesis. RNA isolations were performed in three biological replicates using the SV total RNA isolation kit (Promega, Madison, WI, USA). The quantity and quality of RNA from each sample was measured on a NanoDrop 1000 Spectrophotometer using the absorbance at 260 nm and the A260/A280 ratio, respectively. Subsequently, cDNA was synthesized with the iScript cDNA synthesis kit (Bio-Rad, Hercules, CA, USA) for each replicate within each treatment. Quantitative real-time PCR (qPCR) was performed using iQ SYBR Green Supermix with an iCycler iQ real-time PCR detection system (Bio-Rad). Primers for five *CYP4* genes, *Alpha-tubulin* and the endogenous gene, *Actin*, were used to measure the gene expression of cytochrome *P_450_*, as described in Tiwari et al. [21] (Table 1). Six biological replicates were performed for each treatment. The production of gene-specific products and absence of ‘primer dimers’ was verified by 1% agarose electrophoresis in TAE buffer with ethidium bromide staining.

The 2^−ΔΔ*C*T^ method was used to compare the relative expression of the consensus sequence among PCR products derived from the three dsRNA concentrations and control treatments [34]. This was done by first normalizing the expression level of dsRNA treated samples to *Actin* [21] gene expression, followed by normalization to the treatment giving the lowest gene expression. *Alpha-tubulin* was used as a non-targeted gene (control). Five biological replicates, each consisting of three insects and three technical replicates, were performed for each treatment.

### Western blot assay

Subcellular protein fractions were extracted using the methods described by Wheeler et al. [35] from adults in each treatment. The protein concentration was determined by the Bradford method [36] using a protein assay kit (Bio-Rad Laboratories, Hercules, CA, USA) with ovalbumin as the standard. Since cytochrome P_450_ proteins were clearly detected in the microsomal protein [22], we used the microsomal fractions to perform the Western blot analysis, as described by Tiwari et al. [22].

### Survival assay

The survival assay was carried out on *D. citri* treated with dsRNA-*gfp*, dsRNA- *P_450_*, or RNase-free water as a control. Insects were placed on an autoclaved clear plant tissue culture container (75×75×100 mm) lined with 0.5 mm filter paper saturated with 20% sucrose. Fifty insects were placed per container and five replicates were performed for each treatment. Live insects were counted daily.

### Residual activity of dsRNA- *P_450_*

In order to assess the duration of the RNAi effect, 70 insects treated with RNase-free water or dsRNA-*P450* (20 ng/insect) were placed into plant tissue culture containers with filter paper saturated with 20% sucrose. Samples consisting of three insects were taken daily and kept at −20°C. Cytochrome *P_450_* (general oxidase) activity was measured in all samples as described above. Five replicates were performed for both treatments.

### Pesticide application

To investigate the effect of treatment with dsRNA- *P_450_* on insecticide resistance, *D. citri* adults were treated with the dsRNAs (20 ng/insect) as described above. *D. citri* were initially maintained on Petri dishes with untreated citrus leaf disks for 72 h and thereafter transferred to new Petri dishes that contained leaf discs treated with insecticide solution. Briefly, the leaf disks (60 mm diameter) were excised, dipped in the insecticide solution made in acetone for 30 s, and allowed to air dry in a fume hood for 1 h prior to placement into the Petri dishes as described by Tiwari et al. [26]. We used analytical-grade imidacloprid at the LD_50_ dosage (0.02 ng Al/µl acetone) previously determined by Tiwari et al. [26]. The mortality of *D. citri* adults was assessed after 24 h. dsRNA-*gfp* was used as a negative control for dsRNA- *P_450_*. Fives replicates (Petri dishes), with five insects each, were performed for each of the four *D. citri* populations tested. Each population was subjected to four different treatments: *D. citri* treated with RNase-free water on 1) RNase-free water- or 2) imidacloprid-treated disks, as well as *D. citri* treated with dsRNA- *P_450_* on 3) RNase-free water- or 4) imidacloprid-treated leaf disks.

### Statistical analysis

All analyses were performed using SPSS version 19.0. Survival was calculated during the interval from initial treatment to when all insects died. Overall survival (OS) curves were obtained using the Kaplan-Meier method and comparisons were made using log rank and Wilcoxon tests. Analysis of variance (ANOVA) was used to compare: *i*) Calculated *D. citri* lifespans between various treatments, *ii*) The effect of dsRNA treatments on *CYP4* activity and insecticide resistance, and *iii*) The duration of the RNAi effect. Post hoc pairwise comparisons between treatments were performed with the Tukey honestly significant difference test. Statistical significance was established as *P*<0.05.

## Results

### Treatment with dsRNA-*P_450_* causes down regulation of five *CYP4* genes

**Figure 1:**
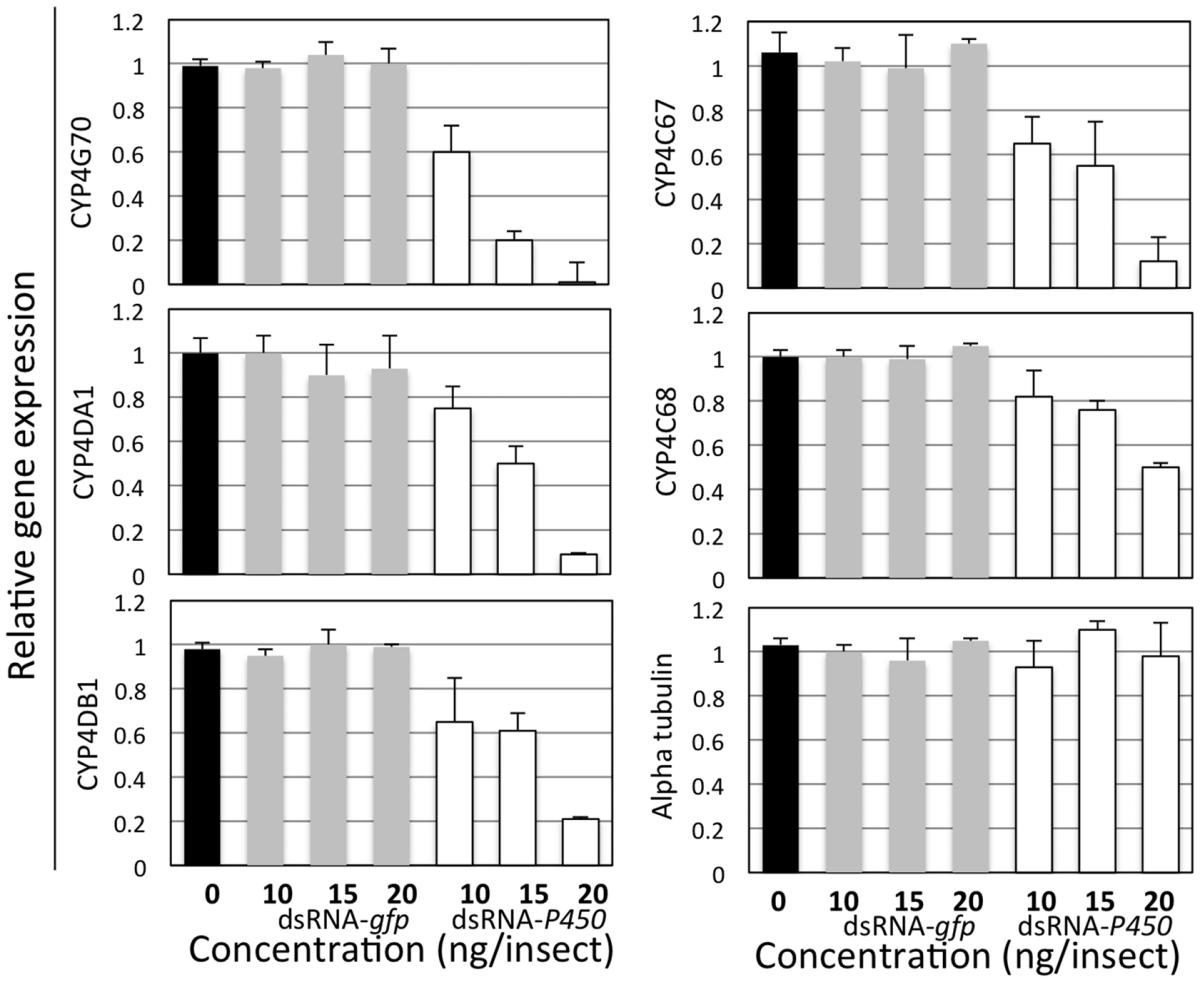
Relative expression levels of the five *CYP4* genes targeted by RNAi in *Diaphorina citri* adults 72 h after treatment with dsRNA. Ct values were first normalized to the endogenous control gene *Actin* followed by normalization to the treatment giving the lowest gene expression using the 2^−ΔΔCT^ method. Standard deviations were calculated based on three independent experiments, each with three technical replicates. Alpha-tubulin was used as a non-target gene control. dsRNA-*gfp* treatment was used as a control targeting an irrelevant gene.

Relative expression levels for the five *CYP4* genes were compared between dsRNA- *P* ***_450_***, dsRNA-*gfp*-treated and control psyllids (Figure 1). Treatment with dsRNA- *P* ***_450_*** caused reduced expression of the five *CYP4* genes. The effect of dsRNA- *P* ***_450_*** was positively correlated with the quantity applied per treatment. The expression level of α-tubulin (non-target gene) remained constant among all treatments indicating the specificity of dsRNA- *P* ***_450_*** to *CYP4* genes. In contrast, there was no effect of the dsRNA-*gfp* control treatments targeting irrelevant psyllid genes on expression levels of *CYP4* genes. The greatest reduction in expression level was found with *CYP4G70*, while the lowest effect was with *CYP4C68*. This observation may help in evaluating gene candidates for RNAi technology for *D. citri*.

### Treatment with dsRNA-*P_450_* reduces the protein expression and the enzymatic activity of *CYP4*

**Figure 2:**
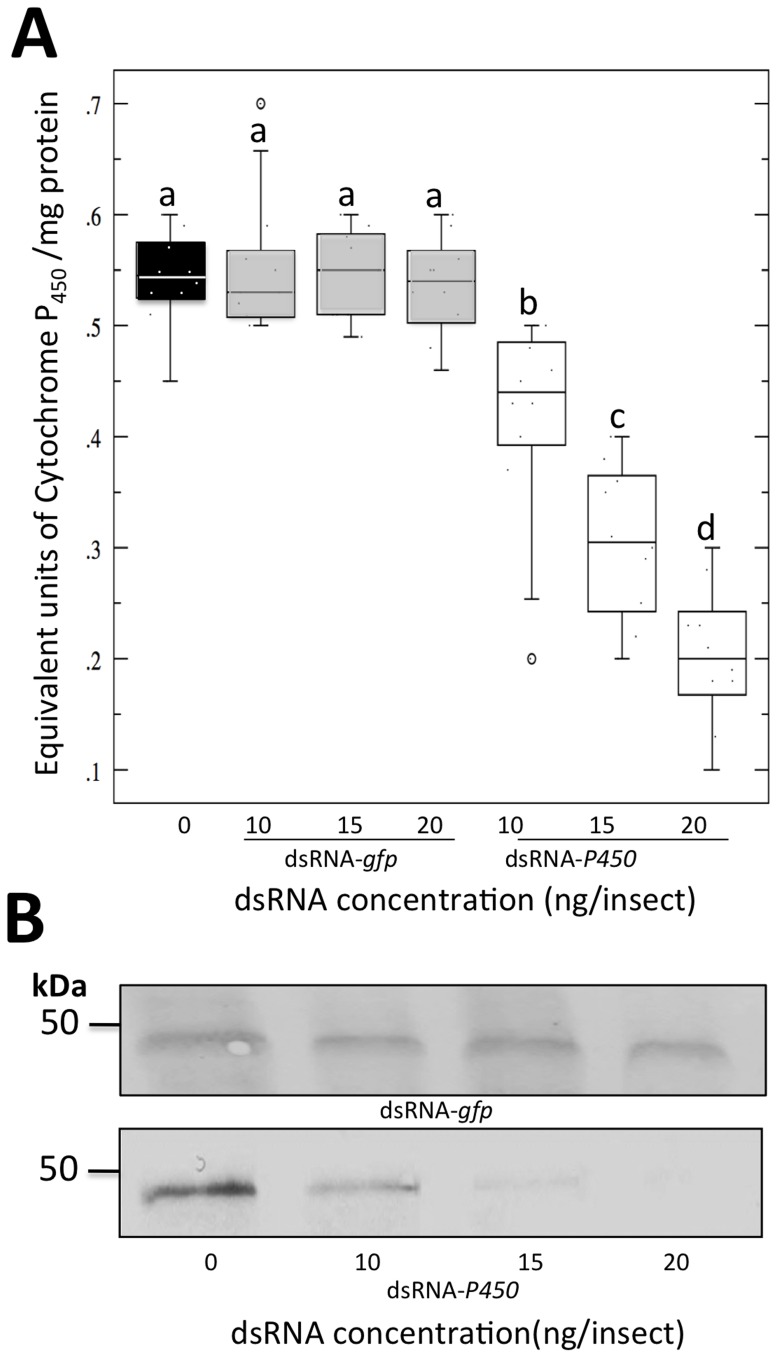
Cytochrome P_450_ (general oxidase) activity and protein expression in dsRNA-treated *D.citri*. A) Box blot representing general oxidase activity. Boxes indicate the interquartile range, including 50% of results, and the mid-horizontal lines represent the median; different letters above deviation error bars represent significant differences between treatments (*P*<0.05). B) Protein analysis of dsRNA-treated and control *D. citri* using Western blot. Western blot was performed using the microsomal proteins prepared from adults treated with three quantities of dsRNA and a control. dsRNA-*gfp* treatment was used as a control targeting an irrelevant gene.

We investigated the effect of treating *D. citri* with dsRNA on general oxidase activity. The activities were similar for all doses of dsRNA-*gfp* treatment, while reduced when *D. citri* were treated with dsRNA-*P_450_*. The activity decreased as the concentration of applied dsRNA- *P_450_* was increased. Additionally, Western blots performed using the microsomal fractions revealed the presence of a band corresponding to a 45 kDa protein that cross-reacted with the primary antibody of cytochrome P_450_ protein. Twenty-five micrograms of microsomal proteins for each treatment were used to perform the Western blot which indicated the highest amount of cytochrome P_450_ proteins (detoxifying enzymes) in *D. citri* treated with 0 ng/µl of dsRNA, followed by *D. citri* treated with 10, 15, and 20 ng/insect of dsRNA (Figure 2). There was no signal detected in *D. citri* treated with 20 ng/insect of dsRNA. Expression levels of target *CYP4* and oxidase activity, as a result of dsRNA treatment, directly correlated with the protein expression in adults treated with dsRNA.

### Silencing of *CYP4* reduced the lifespan of *D. citri*

**Figure 3:**
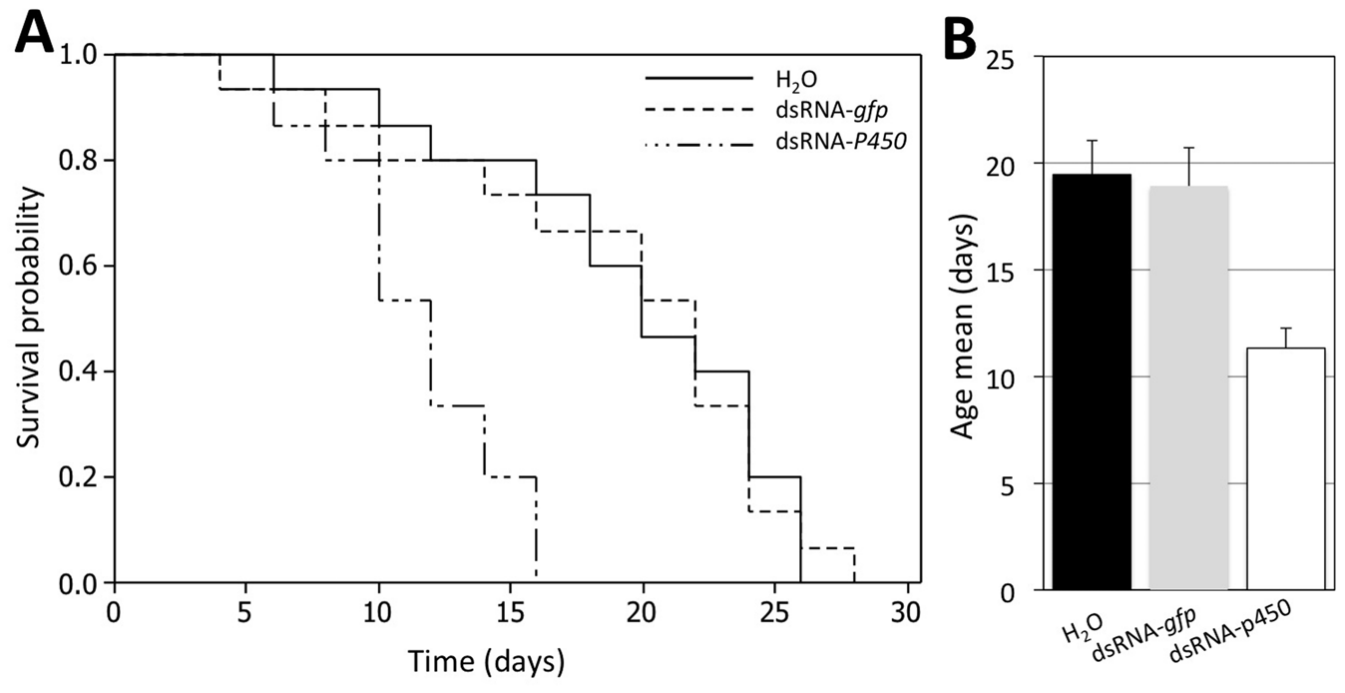
Effect of dsRNA- *P_450_* on *D. citri* survival. A) Kaplan-Meier survival curve showing the effect of dsRNA- *P_450_* treatment on lifespan of *D. citri.* B) Average life span of *D. citri.* Bars represent the standard deviations; different letters above error bars represent significant differences between treatments (*P*<0.05). dsRNA-*gfp* treatment was used as a control targeting an irrelevant gene.

Survival of *D. citri* was quantified following treatment with dsRNA-*gfp*, dsRNA- *P_450_*, and the control (water). The experiment was conducted under the conditions described earlier (Figure 3A). In this experiment, a 20 ng/insect concentration was used for both dsRNA-*gfp* and dsRNA- *P_450_*. A Kaplan-Meier survival plot indicated significant differences among all treatments (log rank = 154.63, *P*<0.001). No significant differences in survival were found between the control and dsRNA-*gfp-*treated *D. citri* (log rank = 1.54, *P* = 0.64). The lifespan in dsRNA- *P_450_*-treated *D. citri* was significantly shorter than that observed for other treatments. Mean lifespans for the treatments are presented in Figure 3B. This suggests that reduced expression of *CYP4* genes shortens the lifespan of *D. citri*.

### Residual activity of dsRNA- *P_450_* treatment

**Figure 4:**
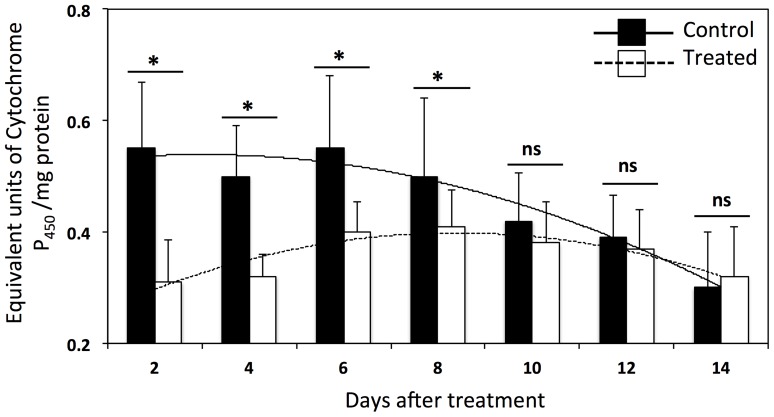
*P_450_* as measured by general oxidase activity. Asterisks indicate significant differences between dsRNA- *P_450_* treated and non-treated *D. citri,* while ns indicates no significant differences(*P*<0.05).

The residual activity of dsRNA- *P_450_* was measured at 20 ng/insect concentration. We used general oxidase activity as an indicator for the residual of dsRNA- *P_450_*. We compared oxidase activity between the control and dsRNA- *P_450_*-treated *D. citri* daily after the treatment application. Oxidase activity was significantly reduced following application of dsRNA- *P_450_* for up to 8 days (Figure 4).

### Silencing of *CYP4* increased insecticide susceptibility

**Figure 5:**
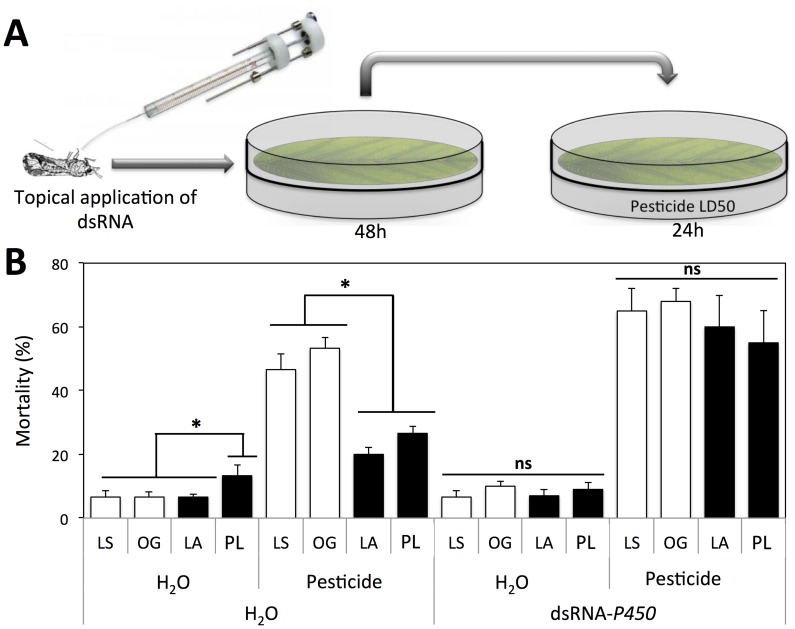
Effect of dsRNA- *P_450_* treatment on *D. citri* susceptibility to imidacloprid using the LD_50_ dosage. A) Illustration of the protocol used to test the effect. B) Percent mortality of *D. citri* after exposure to imidacloprid for 24 h. Insects were maintained on non-treated leaf discs for 48 h after the dsRNA- *P_450_* treatment prior to exposure to imidacloprid. LS: Laboratory susceptible population. OG: Susceptible population collected from an organic grove. PL: Resistant population collected from commercially managed Polk County, Florida. LA: Resistant population collected from commercially managed Lake County, Florida. Asterisks indicate significant differences, while ns indicates no significant differences (*P*<0.05).

In order to determine the effect of dsRNA- *P_450_* on insecticide susceptibility of *D. citri*, we used imidacloprid at the LD_50_ dosage. Two susceptible and two resistant populations were used in this experiment (Figure 5). *D. citri* that were treated with RNase-free water and then exposed to leaf discs treated with imidacloprid exhibited differing susceptibilities, depending on the population tested (Figure 5). Specifically, the two populations from commercially managed citrus groves that had received imidacloprid treatment over the previous several years (LA (Lake County) and PL (Polk County)) were less susceptible to imidacloprid at the LD_50_ dosage than *D. citri* collected from our Laboratory Susceptible culture (LS) and from the Organic Grove (OG) where imidacloprid had not been used previously (Figure 5). Mortality of *D. citri* exposed to imidacloprid after treatment with dsRNA- *P_450_* was increased for each of the four populations as compared with the water control (Figure 5). Mortality of *D. citri* from the two resistant populations (LA, PL), at the LD_50_ dosage of imidacloprid, was significantly higher after treatment with dsRNA- *P_450_* as compared with the water control (Figure 5). Given that dsRNA- *P_450_* also increased mortality of *D. citri* from the two susceptible populations (LS and OG) further suggests that cytochrome P_450_ is implicated in imidacloprid resistance in *D. citri*.

## Discussion

Most insect RNAi studies have relied on the delivery of specific dsRNA through either microinjections [4] or ingestion through feeding [10], [13]. Each of these methods has advantages and disadvantages. The microinjection method requires intense training and is a notably time-consuming technique. In addition, optimization is required for volume selection, place of injection, and needle size for successful dsRNA injection into the insect body [7]. Delivery of dsRNA through ingestion also has limitations, such as reduced effectiveness for inducing RNAi [37], reduced efficacy of dsRNA due to the unfavorable gut environment [38], and difficulties in quantifying the amount of dsRNA ingested [39]. The current work highlights a novel method of dsRNA delivery through topical microapplication to the abdomen of adult *D. citri*. The ventral microapplication allows dsRNA uptake through the exoskeleton of insect. The uptake occurs via the intersegmental membranes. This investigation describes a relatively easy and efficient method for delivering and allowing the dsRNA to enter the insect’s body to induce RNAi. This method has also been described for *D. citri* nymphs with high efficiency of activity [32]. Also, a similar delivery method was reported to induce RNAi in *Ostrinia nubilalis* larvae [11].

A dosage as low as 50 ng/µl of dsRNA down-regulated the expression of the consensus sequence derived from five *CYP4* genes from *D. citri,* as verified by qPCR and Western blot. The lifespan of *D. citri* following dsRNA treatment was statistically shorter as compared with untreated controls in the current investigation. In another example, mortality in *O. nubilalis* larvae ranged between approximately 40–50% following topical treatment with dsRNA [11]. The mortality, coupled with the lack of any other abnormality observed in the dsRNA-treated adult *D. citri*, suggests that the *CYP4* specific dsRNA are highly target specific. Target specificity of dsRNA is also useful considering the potential for dsRNA exposure to non-target organisms under field conditions. Designing target specific dsRNA is not uncommon; species-specific dsRNA has been shown to work like an insecticide by killing specifically targeted insect pests [40]. The low concentrations needed for induction of RNAi and the highly specific nature of dsRNA suggest it might be a tool for managing insecticide resistance in *D. citri*. Our results indicate that dsRNA- *P_450_* reduced oxidase activity, which presumably increased insecticide susceptibility in both resistant and susceptible populations of *D. citri*. In comparison, dsRNA-*gfp* (our negative control treatment) did not affect *CYP4* gene expression or oxidase activity. These findings indicate specificity of RNAi for *D. citri* with the genes targeted in the present investigation.

An important challenge for the application of dsRNA for practical pest control is developing a delivery method for commercial field deployment. Another practical limitation of RNAi that needs to be addressed is that large quantities of dsRNA are expensive to produce. Currently, we are working on inserting the previously described dsRNA into citrus plants for direct ingestion by *D*. *citri* during feeding. Delivery of dsRNA through transgenic plants (Plant mediated RNAi) has been achieved in *Helicoverpa armigera* and *Diabrotica vergifera vergifera* [41], [42]. The absence of interferon-regulated innate immunity pathways in insects allows the possibility of employing longer dsRNA for maximal RNAi [43]. Another potentially feasible way of delivering dsRNA would be to incorporate target-specific dsRNA into bacteria with an appropriate transfection reagent and then spraying the transformed bacteria onto citrus trees. However, future work is needed to evaluate the most efficient transfection reagents and bacterial formulations to prevent the breakdown of dsRNA under field conditions. Once the entire genome of *D. citri* is sequenced, this delivery method could be a convenient way to conduct high-throughput loss-of-function research for determining gene functions. In addition, the current results suggest that further work is needed to understand the mechanism of dsRNA entry into cells following topical application of dsRNA onto *D. citri* to induce RNAi.
